# Inner sense of rhythm: percussionist brain activity during rhythmic encoding and synchronization

**DOI:** 10.3389/fnins.2024.1342326

**Published:** 2024-02-14

**Authors:** Yin-Chun Liao, Ching-Ju Yang, Hsin-Yen Yu, Chiu-Jung Huang, Tzu-Yi Hong, Wei-Chi Li, Li-Fen Chen, Jen-Chuen Hsieh

**Affiliations:** ^1^Institute of Brain Science, National Yang Ming Chiao Tung University, Taipei, Taiwan; ^2^Integrated Brain Research Unit, Department of Medical Research, Taipei Veterans General Hospital, Taipei, Taiwan; ^3^Graduate Institute of Arts and Humanities Education, Taipei National University of the Arts, Taipei, Taiwan; ^4^Center for Intelligent Drug Systems and Smart Bio-devices, National Yang Ming Chiao Tung University, Hsinchu, Taiwan; ^5^Department of Biological Science and Technology, College of Biological Science and Technology, National Yang Ming Chiao Tung University, Hsinchu, Taiwan; ^6^Brain Research Center, National Yang Ming Chiao Tung University, Taipei, Taiwan

**Keywords:** percussionist, percussion training, externally directed cognition, internally directed cognition, encoding, synchronization, functional magnetic resonance imaging

## Abstract

**Introduction:**

The main objective of this research is to explore the core cognitive mechanisms utilized by exceptionally skilled percussionists as they navigate complex rhythms. Our specific focus is on understanding the dynamic interactions among brain regions, respectively, related to externally directed cognition (EDC), internally directed cognition (IDC), and rhythm processing, defined as the neural correlates of rhythm processing (NCRP).

**Methods:**

The research involved 26 participants each in the percussionist group (PG) and control group (CG), who underwent task-functional magnetic resonance imaging (fMRI) sessions focusing on rhythm encoding and synchronization. Comparative analyses were performed between the two groups under each of these conditions.

**Results:**

Rhythmic encoding showed decreased activity in EDC areas, specifically in the right calcarine cortex, left middle occipital gyrus, right fusiform gyrus, and left inferior parietal lobule, along with reduced NCRP activity in the left dorsal premotor, right sensorimotor cortex, and left superior parietal lobule. During rhythmic synchronization, there was increased activity in IDC areas, particularly in the default mode network, and in NCRP areas including the left inferior frontal gyrus and bilateral putamen. Conversely, EDC areas like the right dorsolateral prefrontal gyrus, right superior temporal gyrus, right middle occipital gyrus, and bilateral inferior parietal lobule showed decreased activity, as did NCRP areas including the bilateral dorsal premotor cortex, bilateral ventral insula, bilateral inferior frontal gyrus, and left superior parietal lobule.

**Discussion:**

PG’s rhythm encoding is characterized by reduced cognitive effort compared to CG, as evidenced by decreased activity in brain regions associated with EDC and the NCRP. Rhythmic synchronization reveals up-regulated IDC, down-regulated EDC involvement, and dynamic interplay among regions with the NCRP, suggesting that PG engages in both automatic and spontaneous processing simultaneously. These findings provide valuable insights into expert performance and present opportunities for improving music education.

## 1 Introduction

Percussionists, often considered the rhythmic pulse of musical ensembles, assume a distinct and vital role in establishing the foundational structural framework that unifies diverse musical elements into a coherent whole. Rhythm and tempo, fundamental and interconnected aspects essential to the essence of music, hold a central position in ensuring musical coherence (Wang, 1984; Jones et al., 2010). The steadfast maintenance of rhythm and tempo are paramount for percussionists, prompting them to cultivate an inner sense of rhythm and tempo (Repp and Desain, 2002; McAuley, 2010). This crucial responsibility is deeply rooted in their extensive training, which places a strong emphasis on mastering the intricate rhythmic patterns that underlie tempo control (Cameron and Grahn, 2014). These complex rhythmic patterns not only distinguish their performances but also serve as defining elements of their musical expression (Palmer, 1997).

The neural correlates of rhythm processing (NCRP) involve key brain regions such as the putamen, superior temporal gyrus (STG), pre-supplementary motor area (pre-SMA), supplementary motor area (SMA), primary motor cortex (M1), premotor cortex (PMC), inferior frontal gyrus (IFG), inferior parietal lobule (IPL), superior parietal lobule (SPL), and the cerebellum (Kasdan et al., 2022). These regions play essential roles of rhythm functions in processing sensory perceptions, regulating motor control, perceiving beat perception, and coordinating the timing of sequences (Grahn and Brett, 2007; Chen et al., 2008; Konoike et al., 2015; Manning and Schutz, 2016; Kasdan et al., 2022).

In the context of their musical pursuits, percussionists heavily rely on external stimuli, which encompass rhythmic notations and auditory beats, as pivotal tools in translating their motor skills and bodily movements into the intricate art of rhythmic drumming (Duke, 1994; Palmer, 1997; Brown et al., 2015). This creative process transcends mere physical expression and extends to the meticulous calibration of precise timing and the organization of rhythmic patterns, all serving the purpose of effectively conveying their distinctive artistic interpretation during rhythm performances (Zhukov, 2004). The primary objective for percussionists resides in their capacity to externalize their inner artistic interpretations through the medium of rhythm. In the domain of percussion performance, the hallmark is the seamless integration of external data, specifically rhythmic notations and auditory cues (Duke, 1994). This external information then undergoes cognitive processing to harmonize with internal cues, most notably in terms of tempo control, culminating in the delivery of rhythmic performances that are not only precise but also expressive. One of the paramount challenges faced by percussionists is the consistent maintenance of rhythm and tempo (Repp and Desain, 2002; McAuley, 2010). This endeavor necessitates a dynamic interplay between two fundamental cognitive processes: externally directed cognition (EDC), characterized by active engagement with external sensory stimuli across various modalities, and internally directed cognition (IDC), closely associated with introspection and self-generated thoughts. IDC often finds application during straightforward or well-rehearsed tasks, with a high degree of automaticity (Dixon et al., 2014; Zabelina and Andrews-Hanna, 2016; Ziegler et al., 2018).

The terms EDC and IDC signify the distinction between focusing on external stimuli and directing cognitive processes inwardly, respectively. The brain areas within EDC encompass the primary sensorimotor cortices, dorsolateral prefrontal cortex (dlPFC), frontal eye field, IPL, lateral prefrontal cortex, visual cortex, and other pertinent regions. These structures collectively play essential roles in processing spatial information, integrating sensory inputs, and executing purposeful responses (Dixon et al., 2014). Conversely, IDC often associates with the default mode network (DMN), linked to introspection and self-generated thoughts (Dixon et al., 2014; Zabelina and Andrews-Hanna, 2016) and may come into play during straightforward or well-rehearsed EDC tasks with automaticity (Dixon et al., 2014; Jenkins, 2019). It is noteworthy that the relationship between EDC and IDC can take on different forms, operating independently, in tandem, or even exhibiting a mutually inhibitory dynamic, contingent upon the specific demands imposed by various cognitive tasks (Dixon et al., 2014; Ziegler et al., 2018; Jenkins, 2019). These cognitive processes are linked to distinct neural networks and areas within the brain, each contributing to the intricate web of cognitive control and artistic expression in percussion performance (Chun et al., 2011; Dixon et al., 2014; Zabelina and Andrews-Hanna, 2016; Ziegler et al., 2018; Jenkins, 2019).

The primary aim of this study is to investigate the cognitive processes of highly trained percussionists as they navigate complex rhythms. Our specific focus centers on percussionists’ exceptional proficiency in rhythmic processing in the dynamic interplay among EDC, IDC, and the NCRP. To achieve our objectives, we designed two experiments closely mimicking real ensemble rehearsal scenarios, with a focus on tasks related to rhythmic encoding and synchronization. We reason that percussionist, due to their extensive training in rhythm, may demonstrate a degree of automatization, allowing for the execution of ordinary tasks with reduced cognitive load (Haith and Krakauer, 2018). Automatization could manifest as reduced engagement (e.g., decreased activity) in networks handling rhythmic tasks, accompanied by heightened spontaneity marked by an upregulation of IDC (increased activity in the related brain regions) and a downregulation of EDC (reduced activity in related brain regions). By delving into the cognitive aspects of long-term, professional percussion training, our research has the potential to provide valuable insights for enhancing cognitive abilities within this specialized group.

## 2 Materials and methods

### 2.1 Participants

Initially, we enrolled 43 participants with a background in percussion and 32 participants without musical training. After excluding individuals with brain abnormalities and excessive head movement, we retained 26 participants in each group for the study. Forty-nine participants demonstrated right-handedness (94% of the total participants), 2 were identified as left-handed, and 1 showed ambidextrous tendencies based on the Edinburgh Handedness Inventory (Oldfield, 1971). The percussionist group (PG) consisted of university students majoring in percussion, with an average age of 24.1 ± 3, comprising 11 males and 15 females. The control group (CG), consisting of 26 university students with an average age of 23.7 ± 2.8, maintained an equal distribution of 11 males and 15 females. In this study, the term CG (i.e., the non-musicians) is used to describe individuals who have received less than 3 years of formal music education in specialized programs (e.g., Yamaha Music School). Given that music education is a compulsory part of the curriculum from elementary to high school in Taiwan, it can be reasonably assumed that control participants in the study possess a foundational understanding of music notation and rhythm patterns. Participants in the PG filled out an extensive questionnaire regarding their percussion background, encompassing details such as the duration (years) of percussion training, the number of hours dedicated to weekly and daily percussion practice. PG participants possessed a minimum of 10 years of percussion experience, including involvement in concert performances. In contrast, CG participants had less than 3 years of formal music training. No participants had a history of neurological disorders or were proficient athletes. The study received approval from the local ethics committee of Taipei Veterans General Hospital, and all participants provided written informed consent.

### 2.2 Percussion training questionnaire

The PG participants specialized in percussion as their primary instrument. The survey was designed to explore their musical training experiences and assess the extent and intensity of their percussion practice. In Taiwan, students embarking on music programs typically initiate their musical training in elementary school with a primary instrument, frequently starting with the piano or a string instrument. As they advance through higher grades in elementary or middle school, it is common for them to select one or more secondary instruments, with this study specifically focusing on percussion instruments as the primary emphasis, while the piano or another instrument takes on a secondary role. The survey sought to investigate different aspects of percussion training, including the length of percussion training and the amount of time dedicated to daily and weekly percussion practice.

### 2.3 Stimuli

In this study, a combination of visual and auditory stimuli was employed. The visual component comprised nine rhythmic stimulus materials, a central fixation crosshair, and a drum icon. These nine rhythmic stimuli were presented in a 6/4 time signature, at a tempo of 100 beats per minute (100 BPM), with one beat representing a quarter note, resulting in six beats per measure (see Supplementary Figure S1). The rhythmic stimuli were created by Finale software (MakeMusic, Inc., Louisville, co, USA). The visual cues and instructions were displayed on a screen and projected during the functional Magnetic Resonance Imaging (fMRI) scan. The auditory rhythmic stimuli consisted of a 100 BPM tempo, played at an appropriate volume using Finale software (MakeMusic, Inc., Louisville, CO, USA), with a sampling rate of 44.1 kHz. The volume of the sound beat was individually tailored for each participant to ensure a comfortable listening level. During the fMRI scan, participants were directed to synchronize their tapping with the rhythm using a bimanual fiber optic response pad (Current Designs Inc.) that was compatible with the MRI environment, while simultaneously interacting with the visual stimuli and listening to the auditory beat. The experiment was administered, and the reaction response registered using Presentation software version 0.71 by Neurobehavioral Systems, Inc.

### 2.4 Experimental design of fMRI experiments

**Figure 1:**
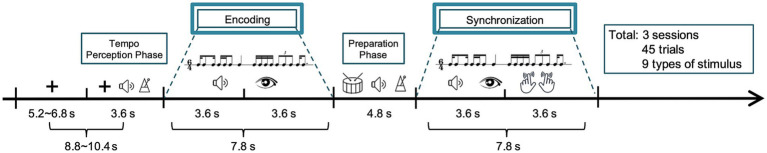
Schematic diagram of the fMRI experiment. The study involved two primary conditions: encoding and synchronization. Participants initiated the process by focusing on a white crosshair for 10.4 s. An auditory beat, maintaining a constant tempo of 100 BPM, began between 5.2 and 6.8 s and persisted for 3.6 s, marking the start of the *tempo perception phase*. Following this, participants observed rhythmic notations in a 6/4 meter, each accompanied by the beat sound, presented twice consecutively, constituting the *encoding condition* (lasting 7.8 s). Following the task, there was a 4.8-s display featuring a drum icon, referred to as the *preparation phase*. In the initial 1.2 s of this phase, a drum icon was presented, followed by the introduction of a tempo of 100 BPM for the remaining 3.6 s. After the beat sound concluded, the rhythmic notations reappeared, presented for two measures consecutively. Participants were then instructed to synchronize their tapping with the 100 BPM tempo in what is designated as the *synchronization condition* (lasting 7.8 s). Each individual trial extended for approximately 30.8 s, and a complete session encompassed 15 such trials, resulting in a total duration of around 462 s. The complete experiment, conducted over three sessions, concluded with a total duration of approximately 1,386 s, equivalent to 23.1 min.

Figure 1 illustrates the experimental setup, which involved tasks of rhythmic encoding and rhythmic synchronization. The fMRI study comprised three scanning sessions. Each task-fMRI session included 15 trials. Before the scanning sessions, participants received task instructions and had the chance to practice tapping the rhythm with both index fingers on a touchpad to become familiar with the tasks within the MRI scanner. During each trial, participants began by focusing on a white crosshair fixation point for a period of 8.8–10.4 s, indicating a rest period. After 5.2–6.8 s, a beat sound started, maintaining a tempo of 100 BPM throughout the 3.6-s trial, referred to as the *tempo perception phase*. Following this, participants viewed rhythmic notations in a 6/4 meter twice, accompanied by the sound beat, for 7.8 s. They were instructed to encode the rhythm during this *encoding condition* (EnC). Following the task, there was a 4.8-s display featuring a drum icon, referred to as the *preparation phase*. In the initial 1.2 s of this phase, a drum icon was presented, followed by the introduction of a tempo of 100 BPM for the remaining 3.6 s. Once the beat sound stopped, rhythmic notations were displayed again. Participants were then required to synchronize their tapping to a rhythm of 100 beats per minute. This activity, termed the *synchronization condition* (SynC), entailed the use of alternating index fingers for tapping on a touchpad. A single trial lasted approximately 30.8 s, and each session, consisting of 15 trials, took around 462 s. The entire three sessions added up to approximately 1,386 s, equivalent to 23.1 min.

To acclimate participants to the experimental setup and confirm their ability to complete the assigned tasks, each underwent a rehearsal phase. This phase included practicing EnC and SynC exercises that were similar to those in the actual experiment, repeated twice before the MRI scans. Importantly, the stimuli used in the rehearsal were not used in the actual experiment to ensure test validity.

### 2.5 MRI data acquisition

All participants underwent imaging using a 3.0 T MRI scanner (MAGNETOM Trio™, Siemens, Erlangen, Germany) located at National Yang Ming Chiao Tung University. The scan employed the head-coil gradient set. Functional images were obtained through T2*-weighted echoplanar imaging (EPI) sequences with the following specifications: 40 slices, 3.4 mm slice thickness, repetition time (TR) = 2000 ms, echo time (TE) = 30 ms, field of view (FOV) = 220 × 220 mm^2^, voxel size = 3.4 × 3.4 × 3.4 mm^3^, matrix size = 64 × 64, repetition number = 228, and a flip angle of 90^o^. Subsequent to the task-fMRI recording, a high-resolution T1-weighted structural image was captured with a resolution of 1 × 1 × 1 mm^3^. T1-weighted 3D structural images were acquired using a magnetization-prepared rapid acquisition gradient echo (MPRAGE) sequence with the following parameters: TR = 2,530 ms, TE = 3.03 ms, slice thickness = 1 mm, field of view (FOV) = 224 × 256 mm^2^, flip angle = 7^o^, matrix size = 224 × 256, and 192 slices.

### 2.6 Image preprocessing

The image data underwent pre-processing using Statistical Parameter Mapping (SPM12, Wellcome Trust Centre for Neuroimaging, University College London, London, United Kingdom^1^) in MATLAB 2022a (The MathWorks, Inc., Natick, MA, USA). Standard pre-processing procedures, including slice timing correction, realignment for head movement correction, co-registration with the T1-weighted image, spatial normalization to the Montreal Neurological Institute (MNI) space using a standard T1 template, and spatial smoothing with an 8 mm Full-Width Half Maximum (FWHM) Gaussian kernel, were carried out. The middle slice of each scan served as the reference slice for slice timing correction, and scans with movements exceeding 3 mm in translation or 3° in rotation were excluded. The normalized T1 images were resampled to isotropic voxels of 2 mm during the normalization process.

### 2.7 Statistical analyses of fMRI data

In first-level analysis, regressors were modeled based on three conditions: fixation, encoding (EnC), and synchronization (SynC). The temporal parameters were defined as follows: the “fix” condition had an onset time at 0 s, lasting approximately 6–7 s; for EnC, the onset occurred around 9 s, with a duration of approximately 7.8 s; and for SynC, the onset was approximately 21 s, lasting about 7.8 s. Multiple regressors, derived from estimated head motion parameters obtained during rigid-body realignment (Van Dijk et al., 2012), were included in the model. The default high-pass temporal filter (cut-off period: 128 s) in SPM was applied to reduce noise. The data underwent regression processing to eliminate non-interest variance sources, incorporating factors such as time derivatives, age, and sex. In second-level analysis, two contrasts were created for EnC and SynC, respectively. Variations between groups in both conditions (EnC and SynC) were investigated through a and two-sample *t*-test within the framework of the random effects model. Analyses were restricted to voxels within the gray matter mask. The threshold was set at an uncorrected voxel level of *p* = 0.001, subsequently adjusted for family-wise error (FWE) correction at a cluster level of *p* = 0.05. Significant activation peaks were identified using the automated antomical labeling (AAL) atlas (Tzourio-Mazoyer et al., 2002). These peaks were then used as seed regions with a 10 mm radius to calculate the percent signal change. Mean activation for each peak was extracted using the MarsBaR toolbox for SPM 12 (version 0.45).

### 2.8 Statistical analysis of demographic data

The Mann–Whitney *U* tests were utilized to evaluate differences in demographic data between groups, with a significance level established at *p* < 0.05 (two-tailed). Statistical analysis was conducted using Statistical Product and Service Solutions 22.0 (SPSS Inc., IBM Corp., Armonk, NY, USA).

### 2.9 Assessment of performance in rhythm synchronization

Performance analysis utilized finger tapping registration during SynC. Each measure was structured with a 6/4 time signature at 100 BPM, lasting 3.6 s. This corresponded to a quarter note at 0.6 s, an eighth note at 0.3 s, a sixteenth note at 0.15 s, and a triplet note at 0.2 s. By subtracting onset time from offset time using finger tapping logs, the range of each measure was determined. These rhythmic intervals were employed to reconstruct the rhythms performed by both PG and CG during SynC.

## 3 Results

### 3.1 Demographic information

Both groups exhibited no notable differences in demographic characteristics, encompassing sex, age, and level of education. On average, PG participants had 18.3 ± 3.4 years of music training and 15.7 ± 3.2 years of percussion training, with a weekly practice of 17.6 ± 9.8 h and a daily practice of 2.6 ± 1.3 h. Furthermore, all PG participants actively participated in concerts. Comprehensive demographic information is available in Table 1.

**Table 1:** Demographic characteristics results.

	**PG**	**CG**	***p-*value**
	**(*n* = 26)**	**(*n* = 26)**	
Age (years)	24.1 ± 3	23.7 ± 2.8	0.73
Education (years)	16.8 ± 1.5	16.1 ± 1.2	0.07
Sex (male/female)	(11, 15)	(11, 15)	1
Duration of music training (years)	18.3 ± 3.4	0.89 ± 1.15	<0.001**
Duration of percussion training (years)	15.7 ± 3.2	–	–
Weekly practice (hours)	17.6 ± 9.8	–	–
Daily practice (hours)	2.6 ± 1.3	–	–

Data expressed as mean ± standard deviation. *p < 0.05, **p < 0.01, ***p < 0.001; PG, percussionist group; CG, control group.

### 3.2 Imaging results

**Figure 2:**
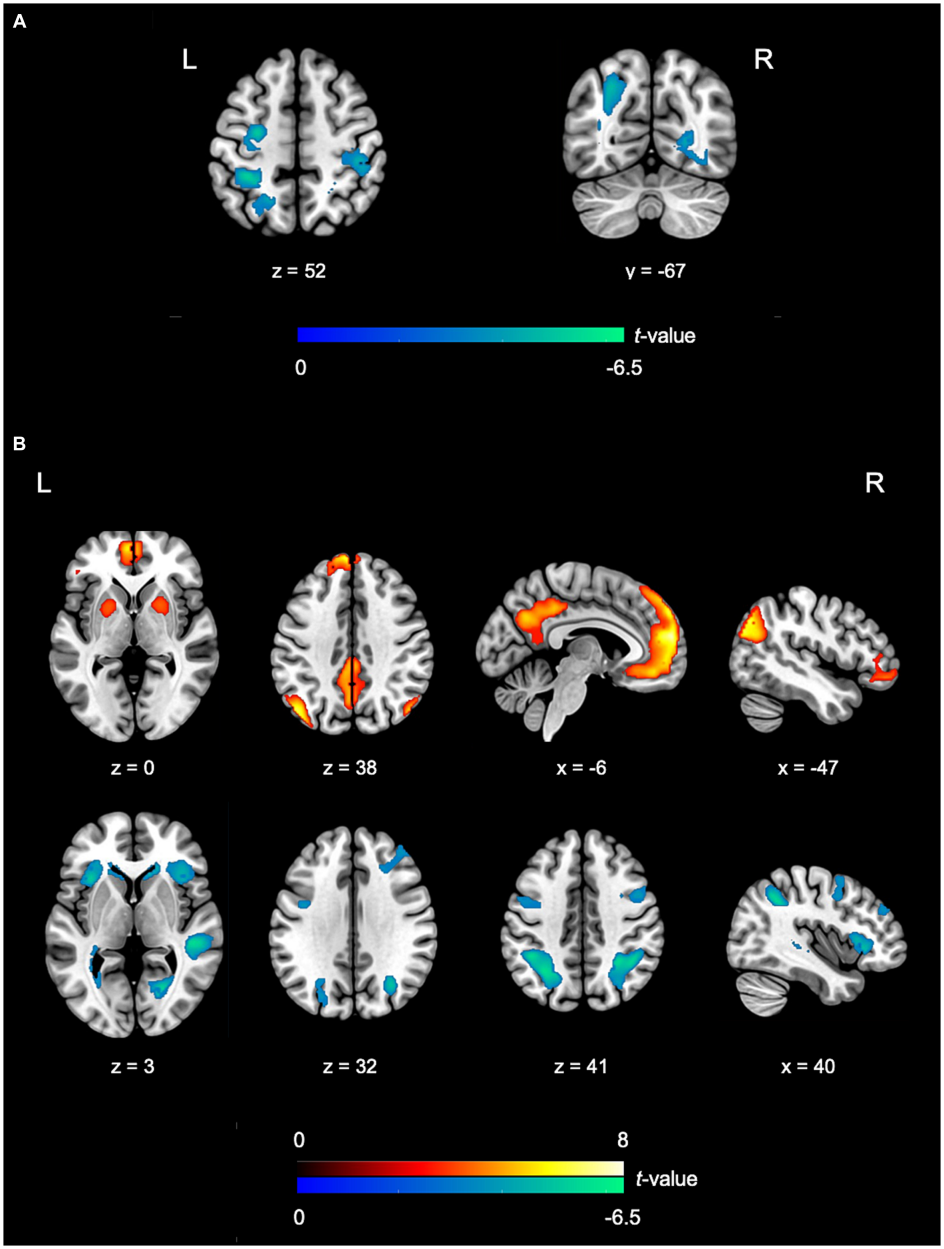
fMRI outcomes depicting the differences between PG and CG in EnC and SynC. **(A)** In comparisons between the PG and CG in the EnC, PG reveals decreased activity in the left dPMC, right postcentral gyrus, right calcarine cortex, left MOG, right fusiform gyrus, left IPL, and left SPL. **(B)** In the comparison between the PG and CG in the SynC, PG exhibits heightened activity in the DMN and diminished activity in the vAI, bilateral dPMC, right STG, bilateral MOG, right calcarine cortex, bilateral IFG, right dlPFC, bilateral IPL, and right SPL. Lower activity is denoted by the blue color, while higher activity is represented by the red color. All visuals adhere to the neurological convention. Further details on significant activity results can be found in Table 2. EnC, encoding; SynC, synchronization; PG, percussionist group; CG, control group; dPMC, dorsal premotor cortex, MOG, middle occipital gyrus; IPL, inferior parietal lobule; SPL, superior parietal lobule; DMN, default mode network; vAI, ventral anterior insula; STG, superior temporal gyrus; IFG, inferior frontal gyrus; dlPFC, dorsolateral prefrontal cortex.

**Figure 3:**
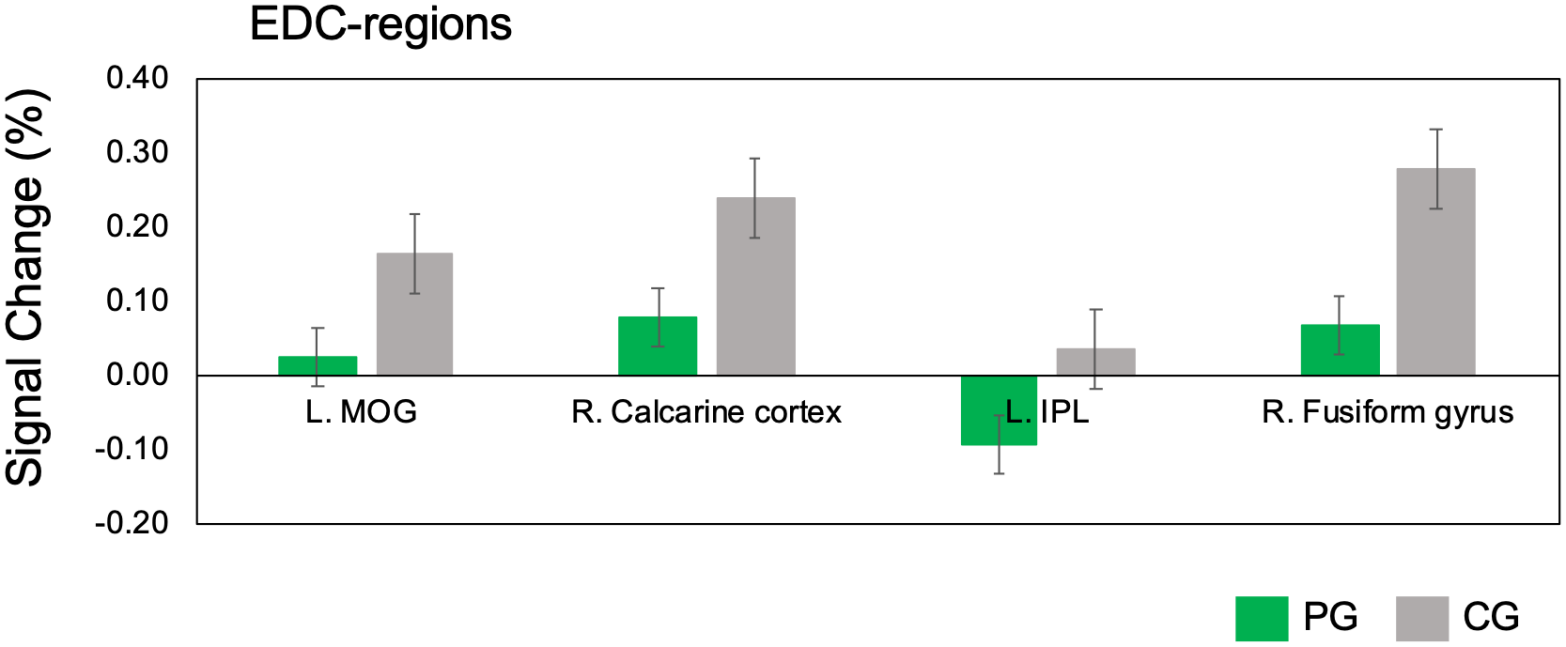
Down-regulation of EDC-regions during rhythmic encoding. The peak signal change is derived from the contrast findings in the between-group comparison conducted during the EnC task. The percentage change in signal, represented by peak values, is extracted from regions centered on the peak coordinates (with a 10 mm radius) using the MarsBaR toolbox for SPM 12. To access comprehensive details about significant results in the EnC, please refer to Table 2. The green bar represents PG, and the grey bar represents CG. Data presented in mean ± SEM. EnC, encoding; SEM, standard error of the mean; L, left; R, right; PG, percussionist group; CG, control group; MOG, middle occipital gyrus; IPL, inferior parietal lobule.

The results of the fMRI data analysis for the EnC and SynC contrasts between the PG and CG are presented in Tables 2, 3, respectively. In the EnC, reduced activity (PG < CG) within EDC-related regions included the right calcarine cortex, left middle occipital gyrus (MOG), right fusiform gyrus, and left IPL (refer to Figures 2A, 3). Moreover, the regions within the NCRP, such as the left dPMC, right postcentral gyrus (specifically the primary somatosensory cortex, S1), and left SPL, exhibited diminished activity. No results suggested increased activation in the PG compared to the CG contrast (refer to Figure 2A).

**Table 2:** Between-group differences in the encoding condition.

**Contrast**	**Region**	**BA**	**Left hemisphere**				**Right hemisphere**			
**Network**			***x***	***y***	***z***	***t*-value**	***x***	***y***	***z***	***t*-value**
***PG > CG***										
	NS	–	–	–	–	–	–	–	–	–
***PG < CG***										
EDC-regions										
	Calcarine cortex	17	–	–	–	–	24	–74	8	–5.37
	MOG	19	–36	–74	12	–5.11	–	–	–	–
	Fusiform gyrus	–	–	–	–	–	30	–72	–4	–4.82
	IPL	40	–36	–40	50	–5.88	–	–	–	–
NCRP										
	dPMC	6	–38	–10	62	–4.5	–	–	–	–
	Postcentral gyrus (S1)	2	–	–	–	–	44	–28	48	–4.94
	SPL	7	–22	–54	48	–4.53	–	–	–	–

PG, percussionist group; CG, control group; BA, Brodmann’s area; NS, not significant; EDC-regions, externally directed cognition related brain regions; NCRP, neural correlates of rhythm processing; MOG, middle occipital gyrus; IPL, inferior parietal lobule; dPMC, dorsal premotor cortex; S1, primary somatosensory cortex; SPL, superior parietal lobule.

**Table 3:** Between-group differences in the synchronization condition.

**Contrast**	**Region**	**BA**	**Left Hemisphere**				**Right Hemisphere**			
**Network**			***x***	***y***	***z***	**t-value**	***x***	***y***	***z***	**t-value**
***PG > CG***										
IDC-regions										
	mSFG	10	−4	58	18	7.82	2	56	2	5.12
	mSFG	9	−6	56	38	6.57	–	–	–	–
	SFG	8	−22	32	54	6.29	–	–	–	–
	AG	39	−46	−74	38	6.8	52	−70	32	6.28
	MTG	21	−58	−14	−14	5.95	–	–	–	–
	PCC	31	−4	−64	34	5.81	2	−62	36	4.99
	Hippocampus	–	−24	−16	−16	5.65	24	−16	−16	5.57
NCRP										
	IFG	47	−48	42	−10	4.75	–	–	–	–
	Putamen	–	−22	6	−6	4.35	22	8	−2	4.16
***PG < CG***										
EDC-regions										
	dlPFC	9	–	–	–	–	30	32	24	−4.64
	STG	22	–	–	–	–	48	−34	6	−5.79
	MOG	19	−26	−76	28	−3.95	32	−72	18	−4.11
	Calcarine cortex	17	–	–	–	–	24	−72	8	−5.46
	IPL	40	−42	−44	38	−6.14	42	−46	38	−5.54
NCRP										
	dPMC	6	−46	0	52	−4.66	46	2	46	−4.69
	vAI	13	−34	20	2	−5.29	36	26	−4	−4.68
	IFG	45	−30	32	4	−4.18	38	22	8	−4.87
	SPL	7	–	–	–	–	26	−70	54	−3.49

IDC-regions, internally directed cognition related brain regions; mSFG, medial superior frontal gyrus; SFG, superior frontal gyrus; AG, angular gyrus; MTG, middle temporal gyrus; PCC, posterior cingulate cortex; dlPFC, dorsolateral prefrontal cortex; STG, superior temporal gyrus; vAI, ventral anterior insula; also refer to Table 2 for other abbreviations.

**Figure 4:**
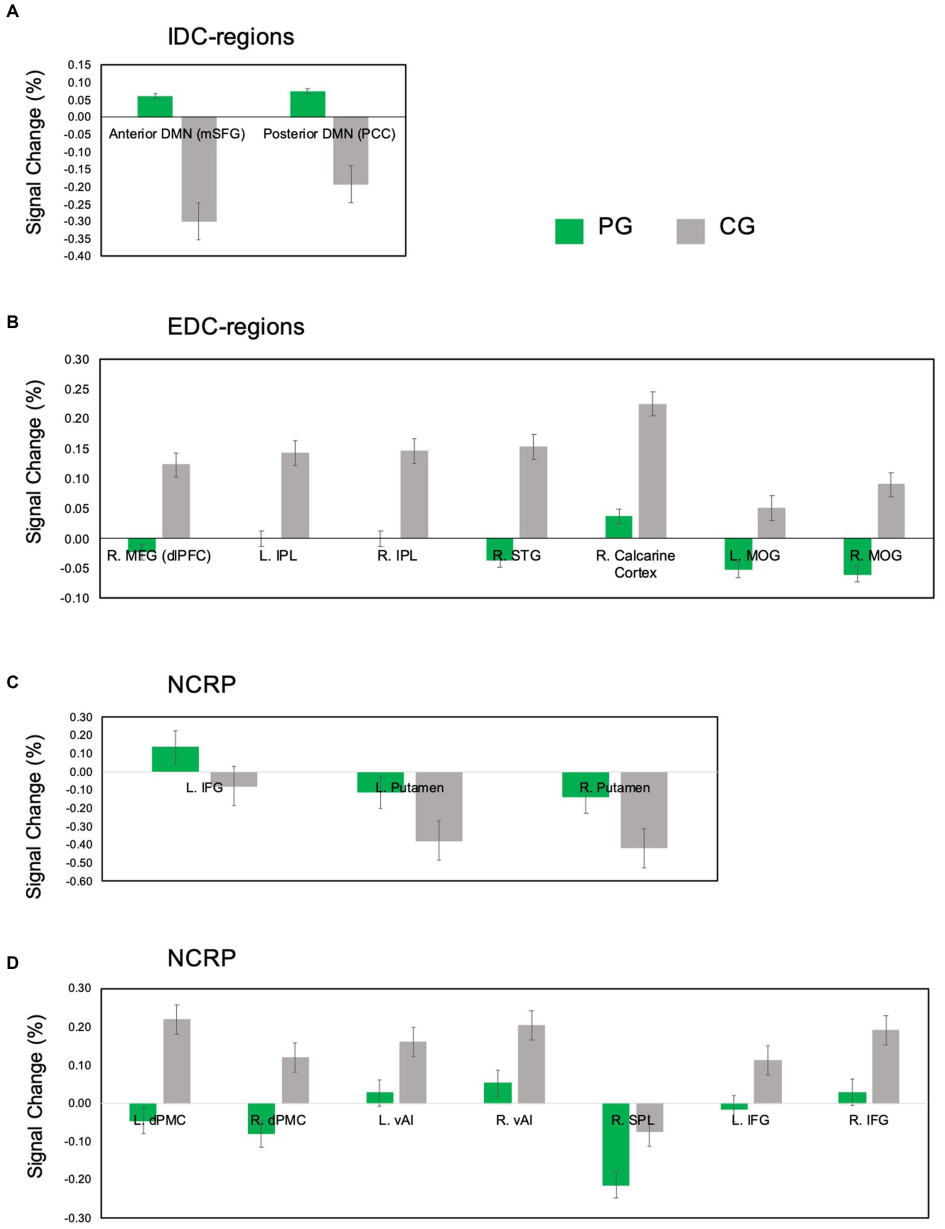
Dynamic regulation of IDC-regions, EDC-regions, and NCRP during rhythmic synchronization. The peak signal change is derived from the contrast findings in the between-group comparison conducted during the SynC. The percentage change in signal, represented by peak values, is extracted from regions centered on the peak coordinates (with a 10 mm radius) using the MarsBaR toolbox for SPM 12. **(A)** Up-regulated IDC-regions; **(B)** Down-regulated EDC-regions; **(C)** Differentially regulated subregions of NCRP. The upper panel shows regions with upregulated activity (increased activity) in PG, while the lower panel displays downregulated activity (decreased activity) in PG. To access comprehensive details about significant results in the SynC, please refer to Table 3. The green bar represents PG, and the grey bar represents CG. Data presented in mean ± SEM. SynC, synchronization; SEM, standard error of the mean; L, left; R, right; PG, percussionist group; CG, control group; IDC-regions, internally directed cognition related brain regions; DMN, default mode network; mSFG, medial superior frontal gyrus; PCC, posterior cingulate cortex; NCRP, neural correlates of rhythm processing; IFG, inferior frontal gyrus; EDC-regions, externally directed cognition related brain regions; dlPFC, dorsolateral prefrontal cortex; IPL, inferior parietal lobule; STG, superior temporal gyrus; MOG, middle occipital gyrus; dPMC, dorsal premotor cortex; vAI, ventral anterior insula; SPL, superior parietal lobule.

Regarding the SynC results, the comparison between PG and CG revealed increased activity in IDC-related areas and specific regions within NCRP, with PG exhibiting greater activity than CG (PG > CG). In contrast, EDC-related regions and NCRP areas showed decreased activity in PG compared to CG (PG < CG) (refer to Figures 2B, 4A–C). The heightened activity in IDC-related regions encompassed the DMN, including the bilateral medial superior frontal gyrus (mSFG), left superior frontal gyrus (SFG), bilateral angular gyrus (AG), left middle temporal gyrus (MTG), bilateral posterior cingulate cortex (PCC), and bilateral hippocampus. Enhanced activation was observed in the left orbital section of the IFG (Brodmann area 47) and the bilateral putamen within the NCRP areas (refer to Figure 4A). Conversely, reduced activity in EDC-related regions involved the right dlPFC, right STG, bilateral MOG, right calcarine cortex, and bilateral IPL (refer to Figures 2B, 4B). Additionally, regions within the NCRP, such as the bilateral dorsal premotor cortex (dPMC), bilateral ventral anterior insula (vAI), bilateral IFG (specifically Boca’s areas, Brodmann area 45), and right SPL, displayed reduced activity (refer to Figure 4C).

### 3.3 SynC performance results

**Figure 5:**
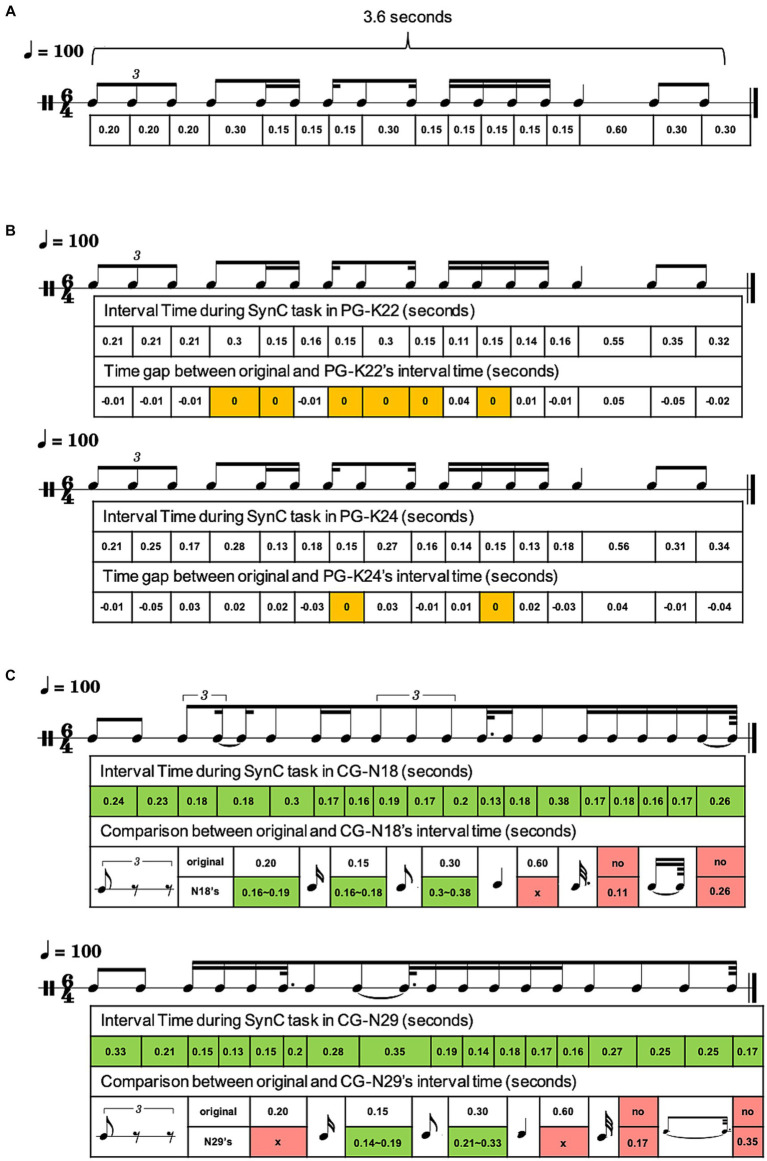
Synchronization outcomes during the SynC. Four instances of rhythmic finger tapping, identified as K22, K24, N18, and N29. In this notation, “K” corresponds to PG, and “N” denotes CG, with the numbers representing the unique participant IDs. **(A)** Provides an overview of the stimulus material used. Each measure adheres to a 6/4 meter with a tempo set at 100 BPM, lasting for 3.6 s. For reference, a quarter note equals 0.6 s, an eighth note is 0.3 s, a sixteenth note measures 0.15 s, and a triplet encompasses 0.2 s. **(B)** Provides a visual representation of the percussion sheet music reconstructed from the recorded key logs played by PG, along with the associated time intervals compared to the reference stimulus. **(C)** Illustrates the reconstructed percussion sheet music derived from the recorded key logs of CG’s performance and the corresponding comparisons to the reference stimulus. These rhythmic notations are created by music notation software. (MuseScore 4.1.1, MuseScore). The color scheme indicates the degree of alignment with the stimulus material: *orange* signifies a perfect match between the intervals played by PG and the stimulus material, *green* highlights substantial discrepancies in CG’s performance compared to the stimulus material, and *red* points out instances of severe rhythm errors in CG’s performances. x, error; NA, not applicable.

The results illustrated PG’s nearly impeccable rhythmic SynC performance, marked by minimal deviations from the reference stimulus material. PG’s renditions consistently exhibited only slight variations in interval timing, and even the triplet notes often displayed negligible differences with a mere 0.01-s discrepancy, occasionally aligning perfectly. In contrast, when assessing CG’s rhythmic SynC against the reference stimulus material, significant disparities were observable. These disparities resulted in performance errors and an incapacity to sustain precise rhythm synchronization throughout the SynC process (see Figure 5 for visual depictions of case presentations). This remarkable ability of PG for rapid and precise rhythmic processing is a testament to the expertise honed through extensive percussion training.

## 4 Discussion

During the EnC, PG experiences a reduction in cognitive load, as demonstrated by decreased brain activity in regions associated with the EDC and NCRP. This reduction implies that less mental effort is needed for task performance. On the other hand, in the SynC, there’s a dynamic interplay among regions within the NCRP, and between brain areas linked to the EDC and those associated with the IDC. This suggests that PG is simultaneously engaging in automatic and spontaneous processing during the SynC.

### 4.1 Lower cognitive effort for PG in encoding the rhythm

**Figure 6:**
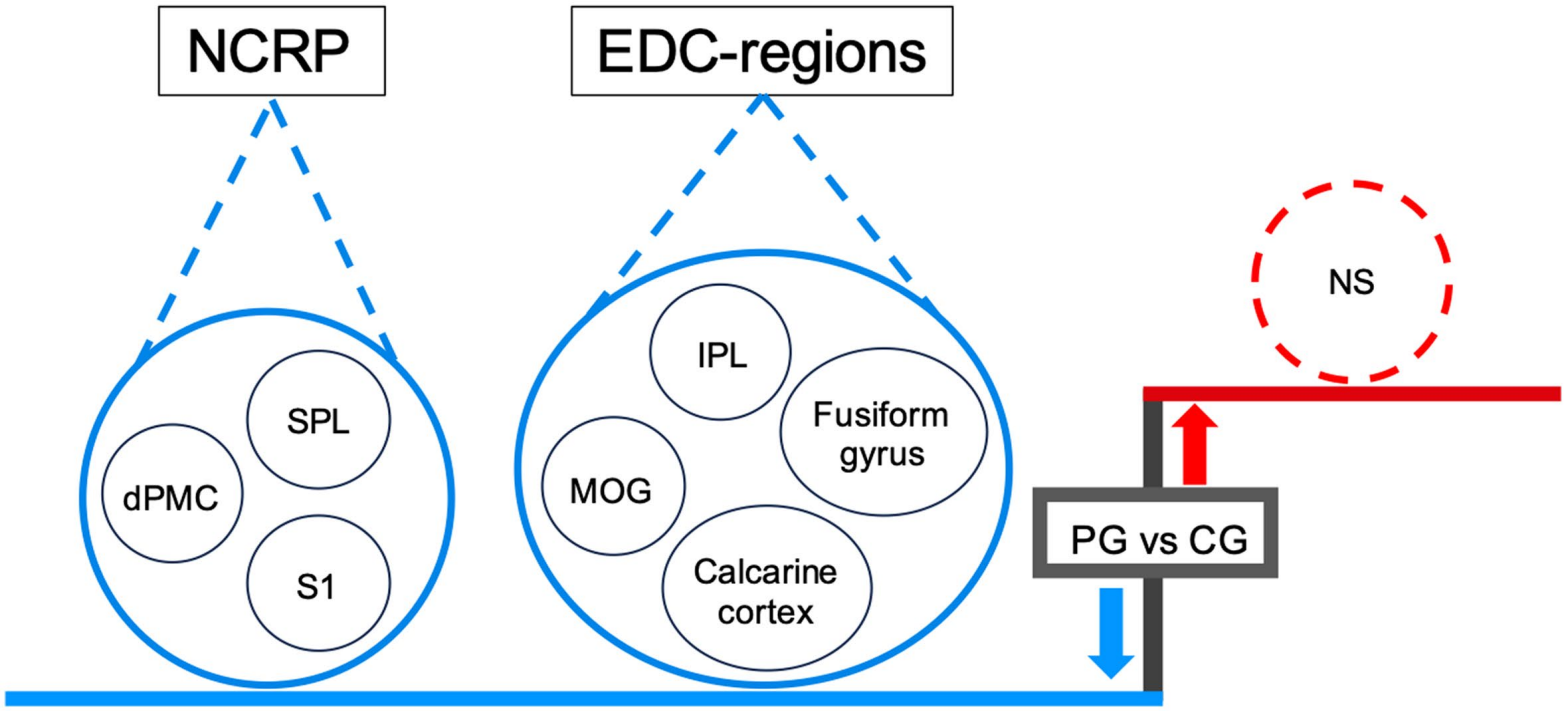
Downregulated EDC and NCRP in PG during rhythmic encoding. The diagram highlights the downregulation of EDC and NCRP in PG, in the context of decreased activity in the relevant regions. The areas are associated with EDC, such as the IPL, fusiform gyrus, MOG, and calcarine cortex, exhibit decreased activity. Likewise, regions within NCRP, such as the SPL, dPMC, and S1, show reduced activity. For statistical details, refer to Table 2. IDC, on the other hand, remains unaltered, with no changes in regional activity. *Blue* color (also downward arrow) indicates decreased activity, *red* color (also upward arrow) indicates increased activity. Abbreviations: EDC, externally directed cognition; NCRP, neural correlates of rhythm processing; IPL, inferior parietal lobule; MOG, middle occipital gyrus; SPL, superior parietal lobule; S1, primary sensorimotor cortex; dPMC, dorsal premotor cortex; NS, not significant.

The EnC, which involves processing rhythmic notes through auditory and visual cues, aligns with the concept of EDC (Chun et al., 2011; Dixon et al., 2014). Compared to CG, the down-regulation of EDC in PG suggests a reduced cognitive load required for the EnC. The diminished activity in EDC-related brain regions suggests that the PG expends less cognitive effort than the CG in organizing information directed towards identical external stimuli (Figures 3, 6).

Furthermore, the PG exhibits reduced activity in motor-related regions (dPMC and SPL) compared to the CG, indicating an enhanced inhibition of motor engagement (refer to Table 2 and Figure 2A). This could be attributed to the increased effort required to suppress the automatic engagement of motor-related regions, even during the mere act of listening to, and observing a virtuoso-related musical piece (Haslinger et al., 2004; Tsai et al., 2010; Petrini et al., 2011).

### 4.2 Diminished cognitive control in PG during rhythmic synchronization

In music performance, synchronization involves aligning with a specific rhythmic template, metronome, or rapid isochronous sequence (Drake and Heni, 2003; Repp, 2006). Performers, during synchronization, must accurately predict action-related patterns, eliciting automatic responses to external cues (Repp, 2006; Keller et al., 2007). The precise determination of “when” and maintaining accurate timing based on external cues is crucial (Carolyn Drakea and Barucha, 2000). As performers gain more experience, automation in their actions increases (Repp, 2006; Vatansever et al., 2017a; Smallwood et al., 2021).

#### 4.2.1 Down-regulated EDC and up-regulated IDC in PG reflecting automaticity and spontaneity

**Figure 7:**
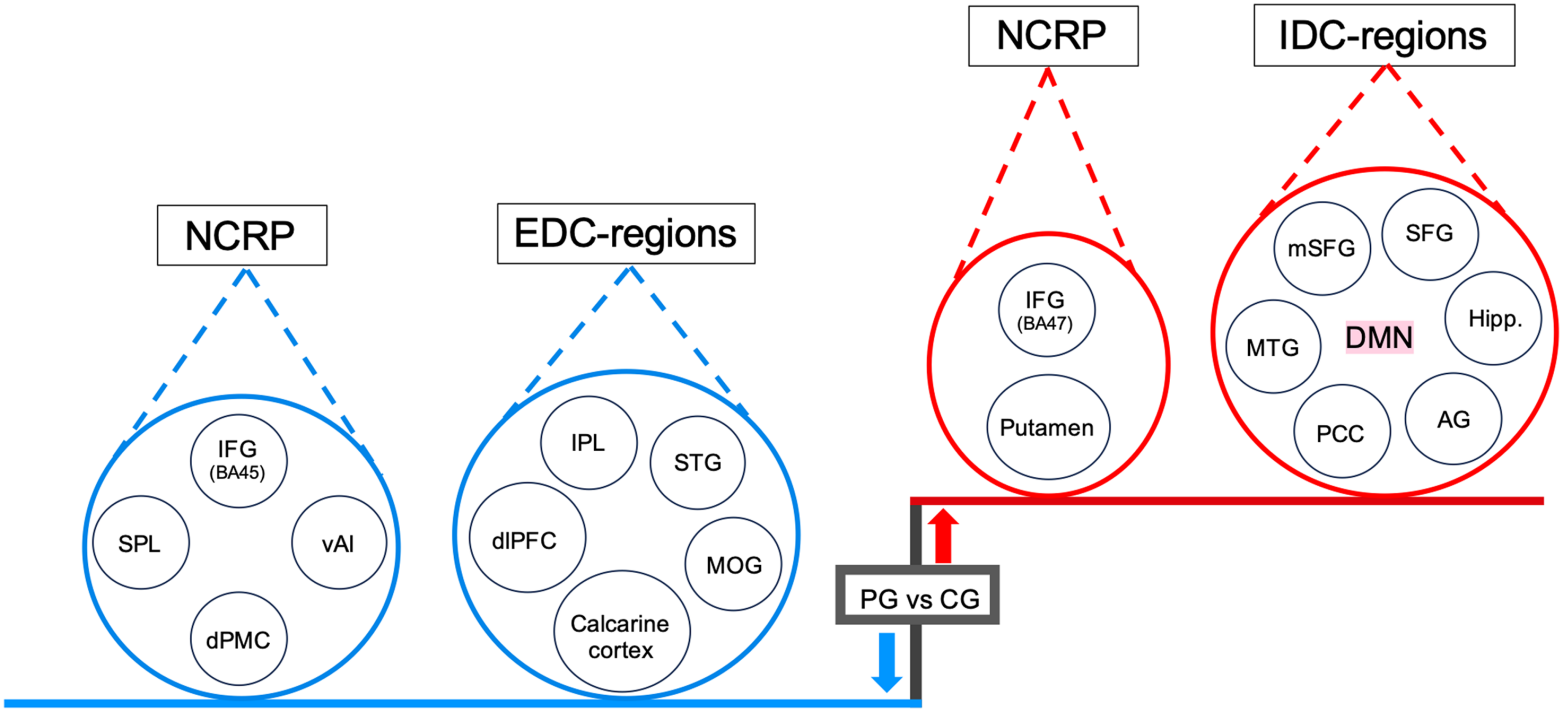
Dynamic interplay between IDC, EDC and NCRP in PG during rhythmic synchronization. The diagram depicts that IDC (the DMN) and certain components of NCRP (the putamen and the IFG, BA 47) exhibit an upregulation in response to increased activity in their respective regions in PG. In contrast, EDC and other components of NCRP show downregulation in response to decreased activity in related regions, which include the IPL, STG, MOG, calcarine cortex, and dlPFC in PG. Additionally, NCRP experiences downregulation in regions like the vAI, dPMC, SPL, and IFG (BA 45). For statistical details, refer to Table 3. *Blue* color (also downward arrow) indicates decreased activity, *red* color (also upward arrow) indicates increased activity. Abbreviations: IDC, internally directed cognition; EDC, externally directed cognition; NCRP, neural correlates of rhythm processing; DMN, default mode network; mSFG, medial superior frontal gyrus; SFG, superior frontal gyrus; Hipp, hippocampus; AG, angular gyrus; PCC, posterior cingulate cortex; MTG, middle temporal gyrus; IFG, inferior frontal gyrus; BA, Brodmann’s area; IPL, inferior parietal lobule; STG, superior temporal gyrus; MOG, middle occipital gyrus; dlPFC, dorsolateral prefrontal cortex; vAI, ventral anterior insula; dPMC, dorsal premotor cortex; SPL, superior parietal lobule.

We observed that PG exhibited lower activity in brain regions associated with EDC and higher activity in brain regions linked to IDC compared to the CG. This observation highlights the dynamic interplay between EDC and IDC in the brain processes (see Figures 4A,B, 7). The activation of EDC-related brain regions, including the STG and dlPFC, signifies engagement in acoustic processing and top-down regulation of temporal sequences in CG (Konoike et al., 2015; Kotz et al., 2018; Kasdan et al., 2022). Musicians acquire proficient techniques through procedural learning, resulting in adaptive behaviors with extended practice (Altenmuller and Furuya, 2016; Tanaka and Kirino, 2016). The dlPFC is known for its role in overseeing higher-order cognitive functions that steer behavior according to internal goals and rules (Koechlin et al., 2003; Hutcherson et al., 2012). Our study observed a decrease in activity within the right dlPFC in PG during the SynC, which indicates a reduction in cognitive control due to adaptive automatization (Miller, 2000; Brovelli et al., 2011). This interaction indicates a nuanced balance between deliberate cognitive control and spontaneous self-evaluative thinking in the PG. Our findings show that the PG leans more toward IDC compared to the CG, engaging in focused internal cognitive processing for precise timing prediction and internal thought. Notably, in the SynC, the PG exhibited heightened activity in the DMN, resulting in more effective decision-making in predictable behavioral contexts and accurate responses due to their extensive percussion training (Vatansever et al., 2017b; Smallwood et al., 2021). This overall down-regulation of EDC and up-regulation of IDC reflects the parallel operation of automaticity and spontaneous processing in rhythmic tasks in the PG.

#### 4.2.2 Decreased activity within NCRP in PG implicating proficiency

Percussionists achieve rhythmic integration and maintain a stable tempo through internalized rhythm processing, utilizing physical movements during performance (Jeannerod, 2001; Kasdan et al., 2022). PG demonstrates decreased activity in the dPMC and SPL, associated with motor cognition, highlighting a lower level of motor control (refer to Table 3 and Figure 2B). Musicians, including PG, refine their motor skills effortlessly through practice, attaining precision and adaptability (Haslinger et al., 2004; Petrini et al., 2011). The acquired proficiency and heightened autonomy in motor skills contribute to a reduction in activation within motor-related regions in PG. Moreover, the vAI, crucial for musicians’ affective processing and subjective evaluation, processes autonomic information and represents emotional experiences (Zamorano et al., 2017; Gujing et al., 2019). When comparing PG to CG during the SynC, reduced vAI activity was observed in PG, suggesting that as musical expertise grows, PG relies more on past experiences than elaborate sensorimotor feedback to assess synchronization in complex rhythmic patterns, possibly associated with heightened autonomic responses in CG (refer to Figure 4C).

#### 4.2.3 Putamen as a neural substrate for inner sense of rhythm

The heightened bilateral putamen activity observed in PG during the SynC could be tied to a robust internal sense of rhythm, as suggested by previous studies (Grahn, 2009; Kasdan et al., 2022). Proficiency in aligning with beats, essential for defining musical tempo and rhythm, is associated with formal musical training (Kindersley, 2012; Spiech et al., 2023). In multitasking scenarios, PG demonstrates the crucial ability to uphold a stable internal beat, particularly important for rhythmic drumming. This stands in contrast to CG, who relies more on external cues for rhythm synchronization.

#### 4.2.4 Distinctive strategic approaches to rhythmic synchronization in PG and CG

Within the left IFG, BA44 and BA45 are linked to syntactical aspects like musical meter, while BA47 is associated with semantic processing (Fadiga et al., 2009; Udden and Bahlmann, 2012; Alfredo Ardila and Rosselli, 2017). In music, syntax encompasses structural composition rules, while semantics involves conveying meaning based on individual interpretations (Koelsch and Siebel, 2005; Koelsch, 2012). The reduced activity in BA45 and increased activity in BA47, observed in PG compared to CG during the SynC, may indicate that PG engages more in semantic processing, whereas CG relies more on syntactic and analytical processing (see Figure 4C).

## 5 Limitations and further consideration

During the initial screening phase, we selected non-musician participants using verbal interviews and questionnaires. Our initial goal was to recruit control group participants who had no music training under specialized music education programs (e.g., Yamaha Music School), beyond what is offered in the general education system. However, in Taiwan, it is exceedingly common for kinder garden and primary school students to enroll in such specialized music programs for a year or two. As a result of this widespread practice, we faced challenges in finding university subjects in the city of Taipei (the capital) who met our original strict criteria. Consequently, we revised our definition of non-musician participants for the control group to include those with less than 3 years of training in such specialized music education programs.

Our study revealed a lack of significant correlation between the duration of music and percussion training and changes in the signal. This absence of connection suggests that extended training years do not necessarily translate into more frequent or intensive practice, nor do they guarantee a higher level of specialization (see Supplementary Figure S2). Instead, the duration of training primarily represents the cumulative effects of long-term training rather than an indicator of achieved performance levels.

Despite the challenges presented by scanning noise during the SynC, the PG demonstrated an exceptional level of precision, as depicted in Figure 4. This extraordinary ability for swift and accurate rhythmic processing in the midst of disruptive scanning noise highlights the expertise cultivated through extensive percussion training. In contrast, the CG’s performance may have suffered due to the interference of scanning noise. In future research, incorporating silent intervals within fMRI scanning sessions (Achim et al., 2021; Ljungberg et al., 2021) could offer a valuable approach for investigating noise-free central processing of EDC and IDC, as well as the central effects of real-time auditory feedback on self-assessment and synchronization adjustment.

In the comparative analyses between the PG and the CG for EnC and SynC, the CG serves as the benchmark for statistical analysis and interpretation in imaging studies. Although the CG participants had standard music education weekly from elementary to high school, the intensity of their musical exposure differs significantly from that of the PG. The cognitive tasks in this study could be challenging for the CG, since they involve focusing on both visual and auditory stimuli, and necessitate either cognitive encoding or synchronous rhythmic production, which are demanding tasks for individuals without music experience. Yet, this level of cognitive effort can be considered typical for individuals without specialized music training.

Additionally, we conducted a thorough visual review of the 1st level analysis results for both PG and CG concerning the EnC and SynC tasks. This review verified that the overall brain activation patterns in both groups are largely similar for each task, indicating that similar neural processes are employed by both groups when performing the same task. The observed differences between the groups can be attributed mainly to neuroplastic changes and adaptive reorganization due to intensive training and practice in percussion. Thus, we suggest that the neuroimaging findings are more likely the result of decreased cognitive load and neural adaptations stemming from extensive percussion training.

Furthermore, handedness significantly impacts brain imaging results. Notable differences in brain structure, especially in the basal ganglia, are evident between right-handed and left-handed people, likely due to differences in motor control (Jang et al., 2017). Given that about 94% of our study’s participants were right-handed, our findings primarily reflect the traits of right-handed individuals.

## 6 Conclusion

PG exhibit reduced cognitive effort when encoding rhythm, as indicated by decreased brain activity in the EDC-related regions and NCRP, in contrast to the CG. This could stem from their proficiency in managing complex stimuli. During rhythmic synchronization, PG reveal a dynamic interplay among regions within the NCRP, and between brain areas linked to the IDC and EDC. They prioritize internal cognitive processes for precise timing prediction, engage the DMN for spontaneous processing (with less DMN deactivation for externally driven processing). The decreased EDC-related brain activity in PG also implies a reduced requirement for top–down control during synchronization. The dynamic display within the NCRP reflects a lower level of motor control and higher level in the internalized rhythmic integration. These findings offer valuable insights into expert performance and have potential applications in music education, cognitive training, and neurofeedback methods to enhance timing prediction and synchronization.

## Data availability statement

The raw data supporting the conclusions of this article will be made available by the authors, without undue reservation.

## Ethics statement

The studies involving humans were approved by Institutional Review Board of Taipei Veterans General Hospital. The studies were conducted in accordance with the local legislation and institutional requirements. The participants provided their written informed consent to participate in this study.

## Author contributions

Y-CL: Conceptualization, Formal analysis, Investigation, Methodology, Validation, Visualization, Writing – original draft, Writing – review & editing. C-JY: Conceptualization, Investigation, Methodology, Validation, Visualization, Writing – review & editing. H-YY: Resources. C-JH: Methodology. T-YH: Investigation. W-CL: Investigation. L-FC: Conceptualization, Funding acquisition, Methodology. J-CH: Conceptualization, Funding acquisition, Methodology, Project administration, Resources, Supervision, Writing – review & editing.

## Footnotes

^1^https://www.fil.ion.ucl.ac.uk/spm
