# Supplementary material for: Inner sense of rhythm: percussionist brain activity during rhythmic encoding and synchronization
Source: Front Neurosci. 2024 Feb 14;18:1342326. doi: 10.3389/fnins.2024.1342326 (PMC10899486; doi:10.3389/fnins.2024.1342326)
Supplement: Supplementary file 1 [file Data_Sheet_1.PDF]

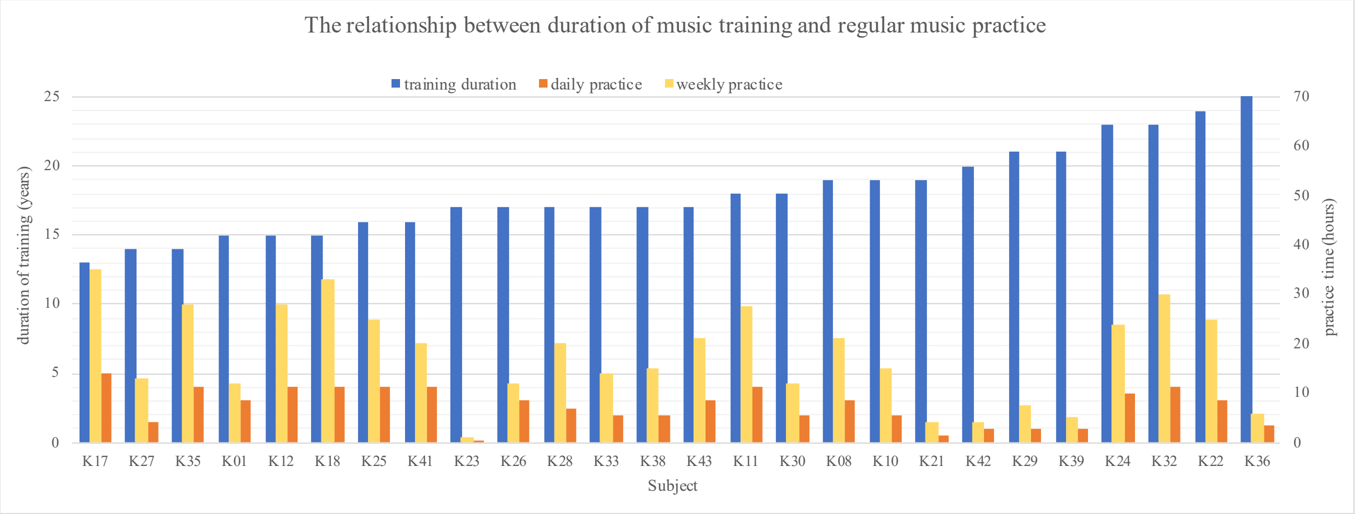

Supplementary Figure 2. The relationship between training duration and practice intensity. The dual-axis chart illustrates the dissociation between training duration (measured in years) and practice intensity (measured in hours per day or week) on distinct scales. The figure demonstrates that a longer training duration does not correspond consistently to higher practice intensity. The x-axis represents each percussionist. The left y-axis represents years of training duration, while the right y-axis represents hours of practice time per week. The blue bar graph represents years of music training, arranged in descending order. The yellow bar graph represents the average practice time per week, while the orange bar graph represents the average practice time per day.
