# Supplementary material for: Inner sense of rhythm: percussionist brain activity during rhythmic encoding and synchronization
Source: Front Neurosci. 2024 Feb 14;18:1342326. doi: 10.3389/fnins.2024.1342326 (PMC10899486; doi:10.3389/fnins.2024.1342326)
Supplement: Supplementary file 2 [file Data_Sheet_2.PDF]

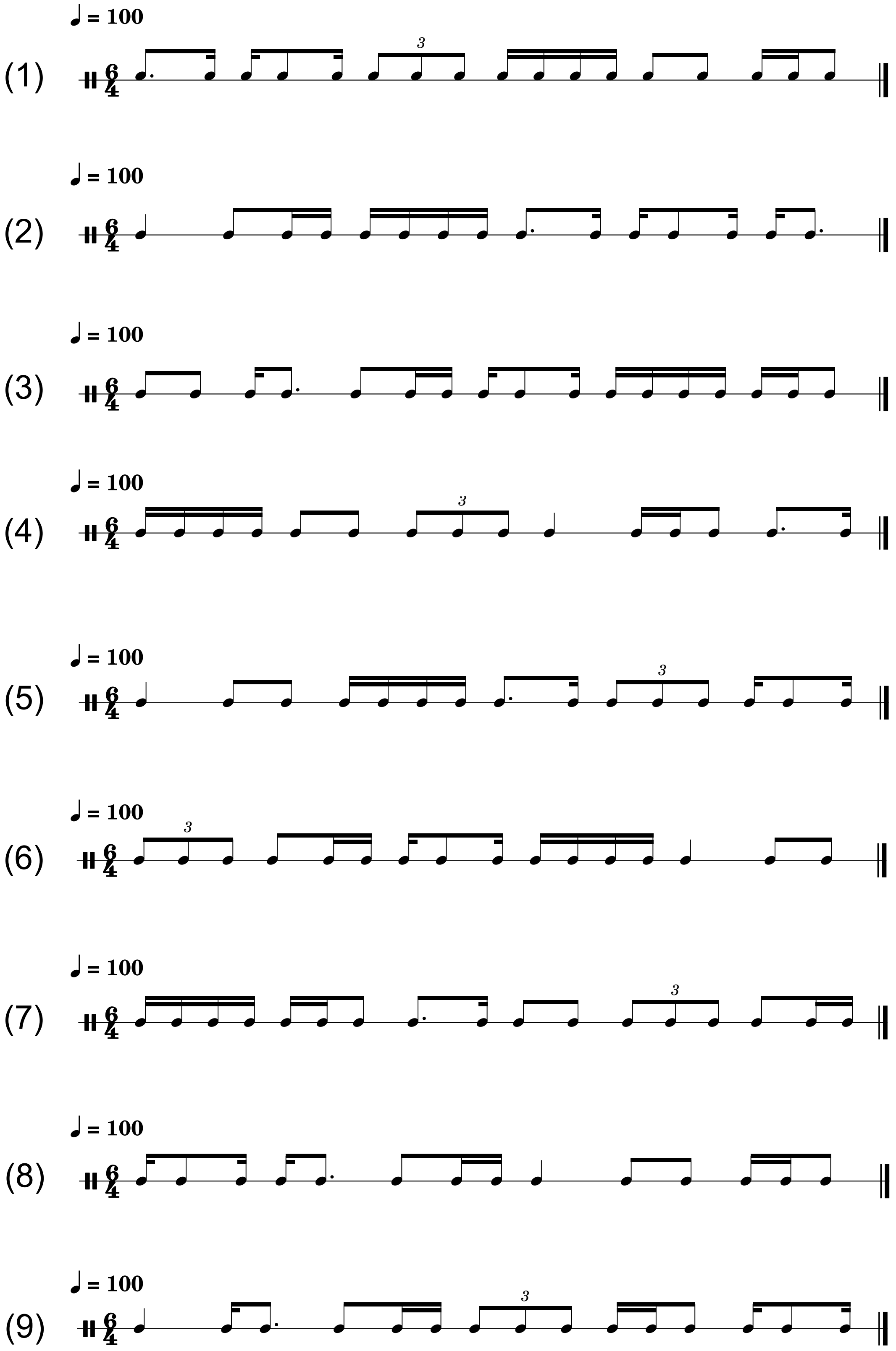

Supplementary Figure 1. Visual stimuli of rhythmic notes. These nine stimuli are presented within a 6/4 meter at a tempo of 100 BPM, where one beat corresponds to a quarter note, and each measure comprises six beats. The stimuli encompass quarter notes, eighth notes, sixteenth notes, and triplet notes. BPM, beat per minute. The rhythmic stimuli are created by music notation software (MuseScore 4.1.1, MuseScore).
